# Dyspnea, the silent threat in Parkinson’s: a mixed methods study

**DOI:** 10.1186/s12883-024-03608-0

**Published:** 2024-07-01

**Authors:** Aseel Aburub, Mohammad Z. Darabseh, Zaina E. Abu-Khdair, Mohannad A. E’layan, Tariq Al Aqqad, Sean J. Ledger, Hanan Khalil

**Affiliations:** 1 Department of Physiotherapy, Faculty of Allied Health Sciences, Applied Science Private University, Amman, Jordan; 2 Department of Physiotherapy, University of Jordan, Amman, Jordan; 3 Department of Physical Therapy, Fatima College of Health Sciences, Abu Dhabi, United Arab Emirates; 4 Physical Therapist, Arab Medical Center, Amman, Jordan; 5 Ministry of Health, Amman, Jordan; 6 Head of Physiotherapy, School of Health, Medical and Applied Sciences, College of Health Sciences, CQUniversity, Rockhampton North, Australia; 7 Department of Rehabilitation Sciences, College of Health Sciences, Qatar University, Doha, Qatar

**Keywords:** Dyspnea, Parkinson’s, Shortness of breath, Respiratory function, Respiratory complication

## Abstract

**Background:**

Dyspnea is considered a silent threat to people diagnosed with Parkinson’s disease and may be a common concern in patients, however, little is known about how it affects quality of life. This study explored the experiences of independently mobile people who are affected by dyspnea in daily life.

**Methodology:**

This was a cross-sectional mixed methods study that included an online questionnaire and semi-structured interviews. The participants were included if they were diagnosed with Parkinson’s disease; had a self-reported Hoehn and Yahr Score I, II or III; were mobilizing independently; and were Arabic speakers. Participants were excluded if they had any other musculoskeletal, cardiac, respiratory, or neurological diseases; or were previous or current smokers; or had been previously hospitalized due to respiratory complications.

**Results:**

A total of 117 participants completed the Arabic version of the Dyspnea-12 Questionnaire. Dyspnea was reported in all participants and that it had an adverse effect on their quality of life, especially during activities of daily living. Additionally, participants reported a lack of knowledge about pulmonary rehabilitation and were unaware of the availability and potential benefits of participation in programs.

**Conclusion:**

Dyspnea was reported in people in the early stages (Hoehn and Yahr Stages I, II, and III) of Parkinson’s disease, and may benefit from routine assessment of lung function, dyspnea management and participation in pulmonary rehabilitation.

## Introduction

Dyspnea or shortness of breath is commonly reported as a subjective experience of breathing discomfort at rest or during exertional activities that consists of qualitatively distinct sensations that vary in intensity [1]. It is commonly associated with respiratory system impairment and malfunction, weakness in the respiratory muscles, or dysfunction of the brainstem ventilatory centers response to chemoreceptors that are present in the carotid and aortic bodies [2]. A recent systematic review identified that people with Parkinson’s disease [3] associated abnormalities in lung function exhibited dyspnea at rest, exertional dyspnea, and daytime somnolence due to hypoxemia [4].

Parkinson’s disease is a progressive extrapyramidal disorder that is characterized by bradykinesia, rigidity, tremor, and impaired postural reflexes [5], with associated non-motor symptoms such as cognitive impairments, neuropsychiatric symptoms, sleep disorders, and respiratory deficits [6]. Respiratory impairments causing Type I or Type II respiratory failure, often leading to pneumonia, are considered the main causes of mortality and morbidity in the advanced stages of the disease (Hoehn and Yahr Stages IV and V) [7, 8] The etiology of dyspnea in Parkinson’s disease is not fully understood but it has been proposed that it involves complex interactions between the cardiovascular and respiratory systems, sensory and perceptual fluctuations, psychological factors, medications and other comorbidities [8]. Other factors that may contribute to the development of dyspnea are biomechanical factors such as kyphoscoliosis, diaphragmatic weakness, reduced lung compliance and chest wall rigidity, which are reflected as a restrictive pattern during lung function tests [9]. These biomechanical factors lead to the stimulation of the stretch receptors of the lungs likely causing increased awareness of the sensation of dyspnea [10]. Decreased levels of dopamine affecting the carotid glomus cells has also been proposed as a contributing factor in dyspnea in Parkinson’s disease [11].

Little is known about the impact of dyspnea on the quality of life of individuals in the early stages of Parkinson’s disease (Stages I, II, and III in Hoehn and Yahr [7]). Understanding how dyspnea affects quality of life whilst individuals are still independently mobile (i.e., those who do not require assistance when walking) is important as it may help clinicians identify strategies to optimize the clinical management of respiratory impairment before the complexities of the latter stages of the disease manifest. Therefore, this study aimed to explore the experiences of independently mobile people living with Parkinson’s disease who were experiencing dyspnea and evaluated its impact on their quality of life.

## Method

### Study design

A cross-sectional mixed-methods study was conducted and included an online questionnaire and semi-structured interviews with people with Parkinson’s disease who reported experiencing dyspnea.

### Ethics

Ethical approval was granted by the Institutional Research Committee at Al-Ahliyya Amman University (AAU/2/8/2022–2023).

## Part 1: online survey questionnaire

### Population, sample, and recruitment

The online questionnaire link was distributed using Microsoft Forms (Redmond, Washington, USA) to members of online social media platforms for Parkinson’s disease (i.e. Facebook, Instagram, and Twitter) who were located in Jordan and the United Arab Emirates (UAE). The inclusion criteria were, (1) a confirmed diagnosis of Parkinson’s disease (Hoehn and Yahr Score is I, II or III [7]), (2) the ability to mobilize independently, and (3) were Arabic speakers. Participants were excluded if (1) they replied “no” to being independently mobile, or (2) they had any other diagnosed musculoskeletal, cardiac, respiratory, or neurological diseases, or (3) were previous or current smokers, or (4) were previously hospitalized due to respiratory complications. A power calculation determined that a sample size of *n* = 106 participants was required for the online questionnaire. Data collection for the questionnaire was conducted between 1st of February 2023 and the 29th of April 2023.

### Survey and data collection

The validated Arabic version of the Dyspnea-12 Questionnaire was completed [12], which included 12 items that assessed the quality of dyspnea symptoms and the severity and emotional response to those symptoms using a 4-point scale (0 = non; 1 = mild; 2 = moderate; 3 = severe).

### Data analysis

Statistical analyses were performed using SPSS 24.0 (IBM Corporation, NY, US). Data was assessed for normality with the Shapiro-Wilk tests. Descriptive statistics were used to calculate, describe and summarize the results of the questionnaires and data were presented as mean, standard deviation (SD), frequencies and percentages unless otherwise stated.

## Part 2: semi-structured interviews

Based on the recommendations of Bowling [13] a minimum of 12 participants were needed to ensure that sufficient open-ended data could be collected for thematic analysis and for meaningful conclusions to be drawn. Therefore, all participants were invited to a telephonic semi-structured interview to discuss their perceptions of dyspnea, and those selected were asked the 5 questions shown in Table 1.

**Table 1:** Topic guide for the semi-structured interviews

Questions
Tell me about your shortness of breath?
Please describe when you usually suffer from shortness of breath?
How long have you suffered from shortness of breath?
How does shortness of breath affect your everyday life?
What do you usually do when you feel breathless?

### Data collection

The interviews were conducted, and audio recorded by AA, a physiotherapy researcher, between the 10^th^ of May − 29th June 2023. In preparation for the thematic analysis of the interviews, all the recorded audios were transcribed verbatim. The transcripts were checked for accuracy by two researchers (AA, MZD), and the process of transcription and trustworthiness of the data were conducted according to published recommendations [14].

### Data analysis

Thematic analysis was used based on responses to questions, following the six steps described by Braun and Clarke [15]:1) Familiarization with data: transcribing; 2) generation of initial codes; 3) searching for themes by sorting relevant data together; 4) combining, refining, separating, or discarding initial themes where needed; 5) refining and defining themes and naming the themes; and 6) producing the report. Data were independently coded by two members of the research team using NVIVO ver. 14 (Lumivero, Denver, USA). Discrepancies were discussed and codes agreed, before the two research independently identified themes. Transcripts were carefully and repeatedly read, and interview recordings listened to, so that the full sense of the interview could be considered. Themes and sub-themes were discussed and agreed within the research team, following an interactive process, and over a series of meetings. Finally, the identified themes and corresponding quotations were translated from Arabic into English by an independent native English-speaking translator at Applied Science Private University.

## Results

### Survey findings

A total of 117 (91 males, and 26 females) participants completed the online version of the Dyspnea-12 Questionnaire [12], which was 110.4% of the targeted sample size. Each questionnaire took an average of five minutes to complete and the mean age of participants was 55.8 ± 7.8 years old. The mean score of the Dyspnea-12 Questionnaire was 24.1 ± 11.1 (range, 0–36) (Table 2).

**Table 2:** Characteristics of the participants who were interviewed

Participant	Age in years	No. years since diagnosis	Dyspnea-12 Questionnaire Score
1	53	5	30
2	49	2	28
3	55	5	30
4	61	7	31
5	72	15	27
6	62	9	28
7	47	2	30
8	60	10	24
9	51	12	29
10	45	1	30
11	62	2	22
12	53	4	31
Mean ± SD	55.8 ± 7.8	6.2 ± 4.5	28.3 ± 2.8

Data of the questionnaire were normally distributed. Responses to the “physical” items showed that 47% of the participants (*n* = 55) reported that they felt short of breath, 56% (*n* = 65) reported that they felt they could not get enough air, 73% (*n* = 85) reported that their breathing was uncomfortable, and 51% (*n* = 43) reported that breathing was exhausting. Responses to the “affective” items of the questionnaire showed that 32% (*n* = 27) of participants reported that breathing made them feel depressed, 19% (*n* = 16) reported that breathing made them feel miserable, 28% (*n* = 24) reported that breathing was distressing, 37% (*n* = 32) reported that breathing made them feel agitated, and 49% (*n* = 42) reported that breathing was irritating.

### Interviews findings

Twelve participants were selected and interviewed; five participants were interviewed telephonically, and seven were interviewed using an online teleconferencing platform. The first two interviews were discussed between the research team to ensure that the described methodology had been adhered to. No concerns were identified, and the remainder of the participants were interviewed. Each interview took approximately 1-hour to complete using the iterative topic guide.

**Fig. 1:**
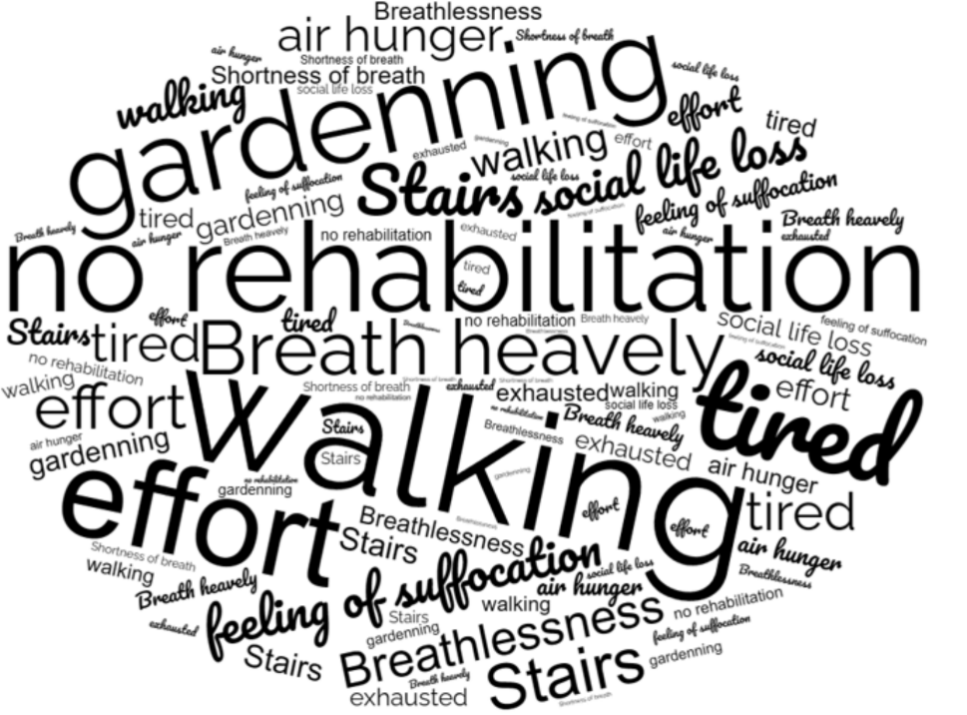
Word cloud of the most used repeated words during the interviews

The transcription and analysis of the interviews were conducted on completion of all interviews. Four themes were identified from the interview data: (1) effects of dyspnea on ability to breathe normally; (2) effects of dyspnea on quality of life; (3) dyspnea easing factors; and (4) knowledge of pulmonary rehabilitation. Figure 1 illustrates a world cloud of the most repeated words during the interviews. Triangulation of data was not undertaken, however as responses to the 5 questions posed to participants were similar, saturation was likely achieved.

### Theme 1: effects of dyspnea on ability to breathe normally

Participants reported that dyspnea made them feel that breathing required significant effort and also identified that taking a deep breath was difficult.

*“I feel I cannot breathe easily! Breathing requires effort, and I do not think that I can take a deep breath. It think it is has been 10 years since I was able to take a deep breath ” (P4).*

Participants reported that they experienced muscle rigidity and loss of elasticity of the chest wall and that this made breathing difficult, because the sensation was similar to their previous experience of dyspnea caused by moderate to intensive exercise..

*“The issue is that I need to have flexible muscles chest muscles to breathe, but mine are not flexible! I feel a tightness in my chest, and I cannot take a breath that inflates or expands my chest! I feel as if my ribs are stiff, and I feel as if I have been running or cycling. I cannot breathe easily” (P7).*

### Theme 2: effects of dyspnea on quality of life

Participants reported that their difficulty breathing impacted their ability to performing activities of daily living, which included reading out loud, walking, showering, gardening, and stair-climbing, which in turn negatively impacted their quality of life.

*“Beside my tremor and rigidity, the shortness of breath limits my ability to take a shower, Additionally, the shortness of breath affects my ability to use the stairs to get to first floor, and I am unable to walk fast” (P9).*

*“I loved taking care of my beautiful olive trees, but now I cannot do so! I cannot do gardening, water the trees, or pick olives like I used to. This is because of both shortness of breath and stiffness in my hands. I feel as if my lungs are asking me to stop gardening. Also, I get breathless when I read out loud or I talking to people” (P1).*

### Theme 3: dyspnea easing factors

Participants reported using bronchodilators and/or adopting positions of ease, such as sitting on a chair, leaning against a wall, or resting on a bed to ease dyspnea.

*“I just sit on a chair and sometimes I lie on my bed when I am breathless” (P11)*.

*“I need to keep walking as I do not want to add breathlessness to my list of problems. I bought an inhaler and this does seem to help the breathlessness, and so I use it when I feel I need to” (P12).*

### Theme 4: knowledge of pulmonary rehabilitation

None of the participants reported that they perform respiratory therapy or engage in pulmonary rehabilitation to alleviate their respiratory symptoms.

*“I do not know what pulmonary rehabilitation is! This is the first time I have heard about it. I barely get physiotherapy for my sore muscles, never mind for my breathing!” (P1)*.

*“Rehabilitation for my lungs? No, I have never heard of it. I only do balance exercises with my physio, but I would love to know about pulmonary rehabilitation. It would be great if I can get my breathing problems under control” (P3).*

Of the 12 participants that were interviewed, only one reported that they knew about pulmonary rehabilitation, but this participant had previously lived in Europe.

*“I know about pulmonary rehabilitation, as I have previously lived in Portugal. We used to do aerobic exercises and chest expansion exercises, and they improved my breathing” (P5).*

## Discussion

This study assessed the effects of dyspnea on quality of life in independently mobile people with Parkinson’s disease living in Jordan and the UAE and assessed their knowledge of the availability of pulmonary rehabilitation. Participants reported that they suffered from the adverse effects of dyspnea in the early stages (I, II and III in Hoehn and Yahr scale) [7]of the disease, and particularly when performing daily activities such as gardening, reading, talking loudly, walking, climbing-stairs, and showering. The study also identified the lack of knowledge about the availability of pulmonary rehabilitation for the management of dyspnea.

Aburub et al. [3] previously reported that people with Parkinson’s disease who lived in the United Kingdom did not complete routine lung function tests or participate in pulmonary rehabilitation, and this was similarly reported in the Jordanian and UAE cohort. Pulmonary rehabilitation has been widely reported to decrease the symptoms of dyspnea in chronic obstructive pulmonary disease [16], amyotrophic lateral sclerosis [17] and interstitial lung disease [18]. However, no randomized controlled trials have evaluated the effects of pulmonary rehabilitation on dyspnea. However, studies have shown the benefits of respiratory muscle training [19] and exercise on exercise tolerance [10] in people with Parkinson’s disease.

There is good evidence of impaired pulmonary function in the early stages of the disease [9, 20] and that these impairments adversely affected quality of life, therefore the recommendation was for routine lung function testing to be initiated. Additionally, it has been suggested that clinicians should actively encourage and promote the potential benefits of participating in pulmonary rehabilitation programs to people with Parkinson’s disease, and especially to those in the early stages of the disease. Clinicians leading these programs should publish baseline and post-effects of the programs on lung function and dyspnea. Physiotherapists that treat patients post exacerbation of respiratory illnesses such as pneumonia, should focus on teaching breathing exercise such as the active cycle of breathing technique to help manage sputum retention and atelectasis. Additionally, lower limb strength training and moderate intensity aerobic exercise should be advocated as these may help to delay or reduce the symptoms of dyspnea.

This study was not without limitations, in that it did not report the Hoehn and Yahr stage of each participant, because the questionnaire was subjective, and the researchers could not depend on participants answers to report accurate scores. This prohibited the analysis of the relationship between the specific disease stage and dyspnea. Future research studies are encouraged to assess the correlations between dyspnea and objectively assessed disease stages. Robust randomized clinical trials that incorporate core pulmonary rehabilitation components of exercise, education, and dyspnea management in people with Parkinson’s disease are warranted.

## Data Availability

The datasets generated and/or analyzed during the current study are not publicly available but are available from the corresponding author on reasonable request.
